# Clinical significance of detecting circulating tumor cells in colorectal cancer using subtraction enrichment and immunostaining-fluorescence in situ hybridization (SE-iFISH)

**DOI:** 10.18632/oncotarget.15452

**Published:** 2017-02-17

**Authors:** Wei Wu, Zhenzhen Zhang, Xian Hua Gao, Zhen Shen, Yan Jing, Haibo Lu, Heng Li, Xiaoye Yang, Xiangbin Cui, Yuqing Li, Zheng Lou, Peng Liu, Cun Zhang, Wei Zhang

**Affiliations:** ^1^ Department of General Surgery, The Aviation Hanzhong 3201 Hospital, Xi’an Jiao Tong University, Hanzhong 723000, Shaanxi, China; ^2^ Zhangjiang Center for Translational Medicine, Shanghai 200120, China; ^3^ Department of Colorectal Surgery, Changhai Hospital, The Second Military Medical University, Shanghai 200433, China

**Keywords:** colorectal cancer, circulating tumor cells, biomarker, recurrence, FISH

## Abstract

Circulating tumor cells (CTC) are useful in early detection of colorectal cancer. This study described a newly developed platform, integrated subtraction enrichment and immunostaining-fluorescence *in situ* hybridization (SE-iFISH), to assess CTCs in colorectal cancer. CTCs were detected by SE-iFISH in 40 of 44 preoperative colorectal cancer patients, and yielded a sensitivity of 90.9%, which was significantly higher than CellSearch system (90.9% vs. 43.2%, P=0.033). No significant association was found between tumor stage, survival and preoperative CTC number. CTCs were detected in 10 colorectal cancer patients one week after surgery; seven patients with decreased CTC numbers (compared with preoperative CTC number) were free of recurrence; whereas two of the three patients with increased CTC numbers had tumor recurrence. Moreover, CTCs were detected in 34 colorectal cancer patients three months after surgery; patients with CTC<2 at three months after surgery had significantly longer Progression Free Survival than those with CTC>=2 (P=0.019); patients with decreased CTC number (compared with preoperative CTC number) had significantly longer Progression Free Survival than those with increased CTC number (P=0.003). In conclusion, CTCs could be detected in various stages of colorectal cancer using SE-iFISH. Dynamic monitoring of CTC numbers could predict recurrence and prognosis.

## INTRODUCTION

Colorectal cancer (CRC) is the fourth common cause of cancer-related deaths in men and the third in women worldwide [1]. Clinical precise management of CRC focus on control of tumor burden and metastasis, and effective treatment is essential to avoid ineffective therapy and unnecessary side effects [2]. Clinically, assessment of treatment responses usually utilizes biomarkers, such as carcinoembryonic antigen (CEA), endoscopy, or serial computed tomography/ magnetic resonance images [3]. The latter is costly procedure and possesses a risk of radiation overexposure, while CEA sensitivity is relatively low (40-60% at the best) [4–6], although detection of changed CEA levels was a reliable predictive factor of prognosis and treatment response [7]. Thus, it is urgently needed to search for tumor markers to monitor treatment responses and predict prognosis of CRC patients.

Blood-circulating tumor cells (CTCs) have been widely investigated in human cancers and demonstrated to play an important role in cancer dissemination. CTCs are tumor cells that have passed the vasculature from a primary tumor site and circulate in the blood stream, first reported in 1869 in a man with metastatic cancer [8]. CTCs can be isolated from the peripheral blood of cancer patients, including CRC patients [9, 10]. The clinical utility of CTC as a marker has been evaluated for both prognosis and prediction of treatment responses in metastatic CRC patients [11]. CTC enumeration detected by CellSearch system was used as a prognostic or predictive biomarker of chemotherapy efficacy in breast, prostate and colorectal cancer patients [12–14]. CTC detected by the CellSearch system has been accepted as a biomarker for breast cancer by the American Society of Clinical Oncology (ASCO) [15]. Principle of the CellSearch system is based on specific combination of antibody to epithelial cell adhesion molecule (EpCAM) on the tumor cell surface, and identification of cytokeratin (CK) in the tumor cell membrane and cytoplasma [16]. However, increasing evidence has emerged that clinical application of the CellSearch strategy is significantly limited because of the inherent methodological deficiency and intrinsic heterogeneity of cell biomarkers in human cancers. In the CellSearch system, CTC capture is to rely on recognition and binding of anti-EpCAM antibody to CTCs; however, EpCAM was reported to be highly heterogeneously and dynamically expressed on the surfaces of many types of cancer cells [17, 18], and only 70% of the examined 134 epithelial solid tumors express EpCAM [19]. CTCs may lose both EpCAM and CKs during epithelial-mesenchymal transition (EMT) [20]. Thus, it is evident that the CellSearch system does have decisive limitations in detection of CTCs [16]. It is therefore imperative to develop an alternative strategy to effectively identify CTCs.

In this study, we utilized a novel strategy integrating subtraction enrichment and immunostaining-FISH (SE-iFISH), which enables effective depletion of WBCs and non-hemolytic removal of RBCs, to establish an expeditious detection of non-hypotonic damaged and non-hematopoietic aneuploid CTCs regardless of EpCAM or CK expression and cell size [16, 21, 22]. Using this approach, we were able to efficiently detect, isolate, and characterize heterogeneous subpopulations of CTC in colorectal cancer patients. Those enriched viable and non-antibody perturbed native tumor cells are suitable for primary cell culture and additional downstream analyses.

## RESULTS

### Patient characteristics

The 44 patients included 26 males and 18 females with median age of 61.5 years old (ranged between 26 and 78years old) and their demographics and clinical characteristics were presented in Table 1. Of the 44 patients, 13 were colon cancer patients and 31 were rectal cancer patients. All of these colorectal cancer patients underwent radical resection of primary tumor lesions (R0) and were histologically confirmed as colorectal adenocarcinoma by postoperative pathological examination. CTCs were assessed using both the SE-iFISH and CellSearch in all of these 44 patients one day before surgery (Preoperative), while CTCs were also detected using SE-iFISH in 10 patients one week after surgery (Postoperative 1 week, P1W) and in 34 patients three months after surgery (Postoperative 3 months, P3M).

**Table 1:** Association of CTCs with clinicopathological parameters

Variations	N	Number of patients		*P*
		CTC-positive	CTC-negative	
Gender				
Male	26	24	2	1.00
Female	18	16	2	
Age, years				
≤60	19	19	0	0.12
>60	25	21	4	
Tumor site				
Colon	13	12	1	1.00
Rectal cancer	31	28	3	
Diameter, cm				
≤4.5	22	21	1	0.60
>4.5	22	29	3	
Differentiation				
Well	3	3	0	0.75
Moderate	39	35	4	
Poor	2	2	0	
Invasion depth				
T1-T2	5	5	0	1.00
T3-T4	39	35	4	
Lymph node				
N0	29	27	2	0.59
N1-N2	15	13	2	
Distant metastasis				
M0	40	37	3	0.32
M1	4	3	1	
TNM				
I-II	28	27	1	0.12
III-IV	16	13	3	
CEA level, ng/ml				
≤5.0	31	29	2	0.57
>5.0	13	11	2	
CA199, U/ml				
≤37	36	33	3	1.00
>37	8	7	1	

### Identification of CTCs in colorectal cancer patients and healthy controls

**Figure 1:**
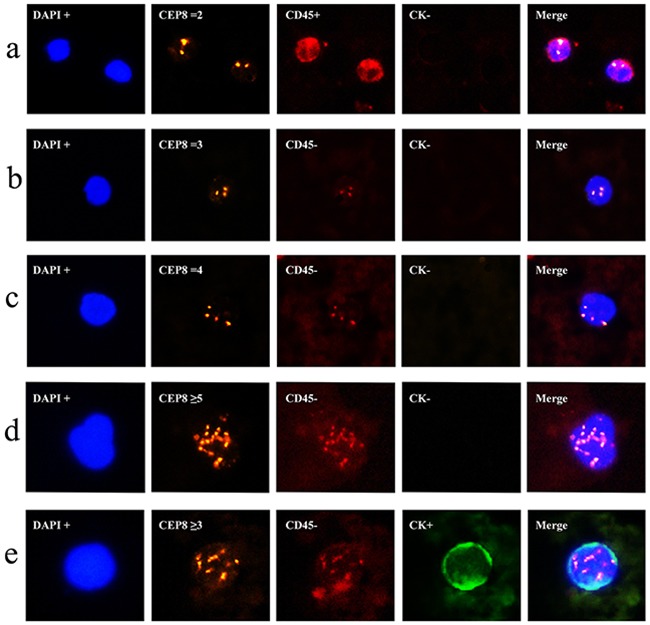
Identification of CTCs in colorectal cancer using the SE-iFISH platform **a**. CK-/CD45+/DAPI+/CEP8=2 (WBC); **b**. CK-/CD45-/DAPI+/CEP8=3; **c**. CK-/CD45-/DAPI+/CEP8=4; **d**. CK-/CD45-/DAPI+/CEP8≥5; **e**. CK+/CD45-/DAPI+/CEP8≥3. DAPI, blue; CEP8, orange; CD45, red; CK, green; iFISH, immunostaining and fluorescence *in situ* hybridization; DAPI, (4′,6-diamidino-2-phenylindole); WBC, White blood cells; CEP8, Centromere Probe 8.

Cells can be recognized differentially by epithelial marker (CK), hematopoietic WBC marker (CD45), existence of cell nucleus (DAPI), or chromosome ploidy (CEP8). These biomarkers had been frequently applied in previous studies of human cancers [16, 23]. In the present study, we adopted these markers to identify CTCs in colorectal cancer. Generally, CTCs were characterized as nucleated cells with epithelial markers and/or hyperdiploid but without CD45 expression (Figure 1). To be more specific, CTCs were defined as CK+/CD45-/DAPI+/CEP8 = 2, CK+/CD45-/DAPI+/CEP8 > 2, CK-/CD45-/DAPI+/CEP8 > 2, whereas CK-/CD45+/DAPI+/CEP8 = 2 was defined as WBC (Figure 1a) [23].

**Figure 2:**
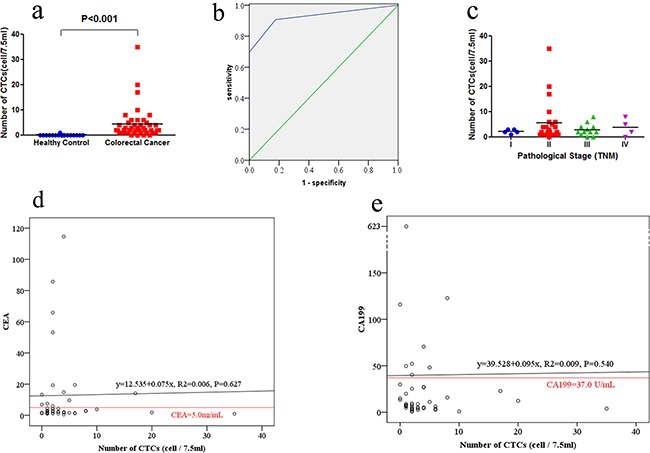
Detection of CTCs in colorectal cancer patients and healthy controls **a**. CTCs enumeration in healthy controls and colorectal cancer. Number of CTCs in 44 colorectal cancer patients and 17 healthy controls were recorded. **b**. The ROC curves for CTCs enumeration to discriminate colorectal cancer patients from healthy controls. The cutoff value was defined as one CTCs in 7.5 mL of blood. **c**. The association of CTCs enumeration with pathological stage (TNM) in colorectal cancer patients. **d**. Comparison of CTC and CEA as blood-based markers. The red horizontal line indicated the CEA threshold of 5.0 ng/mL. **e**. Comparison of CTC and CA199 as blood-based markers. The red horizontal line indicated the CA199 threshold of 37 U/mL.

Using this definition, we detected a total of 189 CTCs in these 44 colorectal cancer patients, of which five were CTC clusters and the remaining 184 were CTC single cell. The features of most CTCs were CK-/CD45-/DAPI+/CEP8>2 (Figure 1b, 1c, 1d), occurring in 40 of these 44 colorectal cancer patients and accounted for 92.1% (174/189) of the whole CTCs, while 15 were CK+/CD45-/DAPI+/CEP8>2 (Figure 1e) in 15 colorectal cancer patients. However, CK+/CD45-/DAPI+/CEP8 =2 and CK-/CD45-/DAPI+/CEP8 = 2 cells were not detected in colorectal cancer. Furthermore, of these 189 CTCs, 92 (in 32 patients) were triploidy (Figure 1b), 30 (in 16 patients) were tetroploidy (Figure 1c) and 67(in 12 patients) were multiploidy (≥5 copies of chromosome 8, Figure 1d and Table 2). In addition, we found one CTC in 17 healthy controls, which was CK-/CD45-/DAPI+/CEP8>2. The CTC number in CRC group was significantly higher than that in healthy control group (Figure 2a).

**Table 2:** Distribution of CK expression and ploidy in the 189 CTCs from 44 colorectal cancer patients

	Number of CTC	CKs	
		Negative	Positive
Diploidy(CEP8=2)	0	0	0
Triploidy(CEP8=3)	92	84	8
Tetroploidy(CEP8=4)	30	27	3
Multiploidy(CEP8>=5)	67	63	4
Total	189	174	15

We then discriminated colorectal cancer patients from healthy controls using the receiver operating characteristic (ROC) curve (Figure 2b). According to Youden's index using a cutoff value of one CTC, 2 CTCs and 3 CTCs in 7.5 mL blood sample yielded sensitivities of 90.9%, 69.8%, 46.5% and specificities of 82.4%, 100% and 100%. Therefore, we defined the cutoff as one CTC in 7.5 mL blood sample (AUC = 0.927). The corresponding Youden's Index is 0.733, sensitivity is 90.9%, specificity is 82.4%, false positive rate is 17.6%, false negative rate is 9.1%, and agreement rate is 91.8%.

### Association of CTC numbers with CRC clinicopathological parameters

The median CTC numbers in 7.5 mL peripheral blood samples were 2 (range, 0-8) and 2 (range, 0-35) in stage I-II and stage III-IV, respectively (P = 0.81; Figure 2c). The positive rate of CTCs were 96.4 % (27/28) in stage I-II and 81.3 % (13/16) in stage III-IV (P = 0.12, Table 1). CTC-positive rates in patients with lower CEA level (≤5 ng/ml) were not significantly different with those having higher CEA level (>5 ng/ml; *P* = 0.57). Similarly, CTC-positive rates among patients with lower CA199 level (≤ 37 U/mL) were not significantly different with those having higher CA199 level (>37 U/ml; *P* = 1.00). In addition, CTC-positive rates were not significantly associated with gender, age, differentiation, tumor position, tumor size, invasion depth, lymph node metastasis and distant metastasis (Table 1).

### Association of CTCs number with serum CEA and CA199 levels from colorectal cancer patients

The median CEA level was 2.72ng/mL (ranged between 0.52 and 114.58ng/mL) and CEA levels were elevated (>5ng/ml) in 13 of these 44 colorectal cancer patients (29.5%). The correlation co-efficiency between CEA and CTC levels was 0.075 (P = 0.627), so CEA and CTC were relatively independent parameters (Figure 2d). The median CA199 level was 8.35U/mL (ranged between 0.8 and 623.1U/mL) and CA199 levels were elevated (>37 U/ml) in 8 of the 44 colorectal cancer patients (18.2%). The correlation co-efficiency between CA19-9 and CTC levels was 0.095 (P = 0.540). Thus, CA199 and CTC were also relatively independent parameters (Figure 2e).

### CK expression on CTC identified by SE-iFISH

Expression of CKs (CK4, 5, 6, 8, 10, 13, and 18) was also assessed on cell membrane and cytoplasma of these CTCs identified by SE-iFISH and showed that CK-positive CTCs were in 10 colorectal cancer patients (10/44, 22.7%). However, CKs-positive CTCs only counted for a relatively small proportion of CTCs (15/189, 7.9%).

### Comparison of CTC enumeration using the SE-iFISH vs. CellSearch

In the CellSearch detecting system, CK(8,18,19)+/EpCAM+/CD45- cells were identified as CTCs. Preoperative CTC enumerations detected by SE-iFISH and CellSearch in 44 patients were shown in Table 3. Specifically, SE-iFISH had a significantly higher positive rate than CellSearch (90.9% vs. 43.2%, P = 0.033) in detection of preoperative CTCs.

**Table 3:** Comparison of SE-iFISH and CellSearch in detecting CTCs enumeration in preoperative colorectal cancer patients (N = 44)

SE-iFISH	CellSearch		Total (%)
	Positive (%)	Negative (%)	
Positive (%)	19 (43.2)	21 (47.7)	40 (90.9)
Negative (%)	0 (0.0)	4 (9.1)	4 (9.1)
Total (%)	19 (43.2)	25 (56.8)	44 (100.0)

### Comparison of preoperative and postoperative one week (P1W) CTC number to predict tumor recurrence

**Figure 3:**
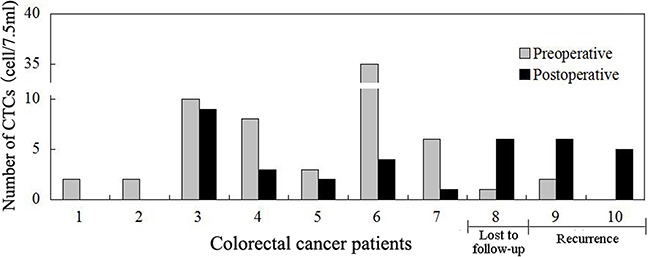
Comparison of preoperative and postoperative one week (P1W) CTCs to predict tumor recurrence in colorectal cancer patients P1W CTC numbers decreased one week after surgery in the first 7 patients who had no tumor recurrence, whereas P1W CTC numbers increased after surgery in the last three patients, two of whom had tumor recurrence three months post-surgery, while another patient lost the follow up.

CTCs were detected in 10 patients one week after surgery (P1W). There is no statistical significance between the preoperative and P1W CTC numbers [(2.5(0-35) CTCs vs. 3.5(0-9) CTCs, *P*=0.44)]. Of these 10 patients, seven with decreased P1W CTC numbers (compared with preoperative CTC) were free of tumor recurrence during the follow up period of time, whereas other three patients with increased P1W CTC numbers had recurrence in two (one with local recurrence and another with intra-abdominal metastasis) and another one lost follow up three months after surgery (Figure 3).

### Postoperative 3 months (P3M) CTC numbers to predict progression free survival (PFS)

**Figure 4:**
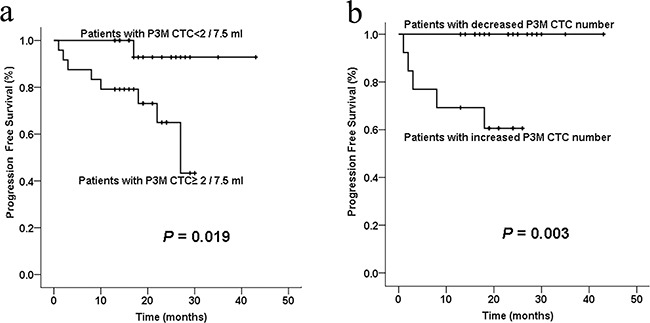
Kaplan-Meier curve analysis of PFS of colorectal cancer patients stratified by postoperative 3 months (P3M) CTC numbers **a**. The average PFS in patients with P3M CTC< 2 was significantly better than those with P3M CTC≥2 (P= 0.019). **b**. The average PFS in patients with decreased P3M CTC number (compared with preoperative CTC number) was significantly better than those with increased P3M CTC number (P= 0.003).

Of these 44 patients, CTCs were detected in 34 patients one day before surgery (preoperative) and 3 months after surgery (P3M). To date, all patients are surviving; thus, we assessed the association of CTC numbers with PFS. Our results demonstrated that preoperative CTC number didn't show statistical significance using the cut-off value of 1, 2, or 3 CTCs per 7.5 ml (P>0.05). However, we evaluated the association between PFS and P3M CTC numbers and found that the median PFS in patients with P3M CTC< 2 was significantly longer than those with P3M CTC≥2 (22.0 vs.17.3months, P= 0.019; Figure 4a). Of the 34 patients, 13 patients had increased P3M CTC number compared with preoperative CTC number, whereas the rest 21 patients had decreased P3M CTC number. The median PFS in patients with decreased P3M CTC number was significantly longer than those with increased P3M CTC number (23.0 vs. 15.3 months, P= 0.003; Figure 4b).

## DISCUSSION

Previous studies showed that CTCs can be frequently detected in peripheral blood of advanced CRC patients [24, 25]. CTC assessment is considered to be a relative noninvasive and sensitive monitoring technique [26]. To date, there have been an increasing number of platforms and techniques to detect and quantify CTCs in different types of cancer. The most common platform used to detect and quantify CTCs in CRC is CellSearch™ system [27, 28], which uses immunomagnetic enrichment of cells expressing EpCAM and CKs. However, the conventional EpCAM-based enrichment and CK-based identification technique has some inherent limitations for sensitivity. In the present study, we applied a newly modified method “SE-iFISH” to detect CTCs in colorectal cancer. It takes advantage of anti-WBC marker antibodies to ensure the depletion of WBCs (as high as 99.99%) and minimum hypotonic injury to CTCs [23]. It can enrich CTCs independent of EpCAM expression and tumor cell size. In the identification process, CEP8, CK, CD45 and DAPI were combined to detect CTCs. Since aneuploidy is a typical common cytogenetic abnormality in tumor cells, this feature could be exploited for CTC detection. Previous studies confirmed that the variation of chromosome numbers could be reflected by CEP8 using iFISH [23]. CTCs can be divided into different subtypes, such as triploidy, tetroploidy and multiploidy based on their chromosomal copy numbers and these subtypes of aneuploidy demonstrated to be significantly associated with the responses of chemotherapy [29]. In our study, all CTCs were polyploidy (CEP8≥3). Of these 189 CTCs identified, 92(48.7%) were triploidy, 30(15.9%) were tetroploidy and 67(35.4%) were multiploidy.

Moreover, cells with characteristics of CK+/CD45-/DAPI+/CEP8=2, CK+/CD45-/DAPI+/CEP8>2, and CK-/CD45-/DAPI+/CEP8>2 were defined as CTCs. It is because positive epithelial and/or hyperdiploid cells are tumor cells in the blood circulation. Cells with characteristics of CK-/CD45+/DAPI+/CEP8 = 2 were WBCs because CD45 is leukocyte-specific transmembrane protein tyrosine phosphatase and diploid of chromosome 8. In the present study, we detected CK expression using the pan-CK antibody, which is broadly reactive with human CK family members (CK4, 5, 6, 8, 10, 13, and 18). We found only 22.7% (10/44) colorectal cancer patients with CK-positive CTCs, which would result in a lower positive rate of CTC if detected by the traditional CK-based system, such as CellSearch. To compare the positive rates of SE-iFISH and CellSearch, we detected preoperative CTC enumerations using these two system in 44 patients and found that SE-iFISH had a significantly higher positive rate than CellSearch (90.9% vs. 43.2%, P = 0.033). In the previous studies, CTC detection rates using the CK-based CellSearch system were between 19.6% and 47.5% [30–35], which is consistent with the positive rate of 43.2% in our study. CKs are most frequently used epithelial markers but could be down-regulated and even missed in the EMT process. This theory could account for the lower positive rate of the CK-based CellSearch system [36].

In the current study, the SE-iFISH achieved a sensitivity of 90.9% and specificity of 82.4% using the cutoff value of one CTC in 7.5 mL blood sample from colorectal cancer. With such a high sensitivity of 90.9%, detection of CTCs using SE-iFISH could be used as an early diagnostic marker for colorectal cancer. The relationship between CTC enumeration and clinicopathological parameters remains unknown. A previous study reported by Sastre et al showed that positive CTCs were only associated with TNM stage, but not with tumor localization, CEA level, and tumor differentiation [37]. But in most published studies in the literature, there was no significant association reported, which may have something to do with the relatively small sample size in these studies [26, 38, 39]. A meta-analysis had collected 646 colorectal cancer patients from nine studies between 1998 and 2006, and demonstrated that positive CTC was significantly related with lymph node metastasis, hepatic metastasis and disease free survival [40]. In our current study, CTC-positive rates were not significantly associated with gender, age, differentiation, tumor position, tumor size, invasion depth, lymph node metastasis, distant metastasis, TNM stage, and CEA and CA199 levels. The small sample size in our study may partly account for this result. The association of CTC enumeration with clinicopathological parameters needs further confirmation in a larger sample size of patients. It was reported that the number and role of CTC differed in colon versus rectal cancer [41, 42]. But no significant difference was identified in CTC number between colon cancer and rectal cancer in our study. Furthermore, it is unreasonable for us to analyze rectal cancer and colon cancer separately due to the relatively small sample size. So, colon cancer and rectal cancer patients were analyzed as a whole in the following analyses in this study.

Previous studies demonstrated that detection of CTC was a novel biomarker in cancer monitoring [29, 43]. In the present study, we detected CTCs one week after surgery (P1W) in 10 patients, and found that P1W CTC enumerations were slightly lower than preoperative CTCs without statistical significance. Of these 10 patients, 7 with decreased P1W CTC numbers were free of tumor recurrence; whereas 2 out of the 3 patients with increased P1W CTC numbers had tumor recurrence. A study reported by Uen et al showed that persistent presence of postoperative CTCs were a prognostic factor in CRC patients who have undergone curative tumor resection [38]. So our data indicated that comparison of preoperative CTCs with P1W CTCs could help medical oncologist to predict CRC recurrence.

We also associated preoperative CTC numbers with PFS of patients, and no significant association was found between PFS and preoperative CTC number, using the cut-off value of 1, 2, or 3 CTCs per 7.5 ml (P>0.05). It may have something to do with the limited sample size and relatively short follow-up. However, the postoperative 3 months (P3M) CTC number did associate with PFS. Patients with P3M CTC< 2 had longer PFS than those with P3M CTC≥2 (P=0.019), while patients with decreased P3M CTC number had longer PFS than those with increased P3M CTC number (P=0.003). CTC number was reported to reflect the chemotherapeutic sensitivity of CRC patients [39], and relate positively with radiographic disease progression in colorectal cancer patients receiving chemotherapy [26, 39]. The postoperative adjuvant chemoradiotherapy usually begins at one month after operation. The P3M CTC number was assumed to be detected after 2 months of chemoradiotherapy, and may become an indicator of chemoradiotherapy sensitivity. Therefore, our study revealed that monitoring of CTC number using this novel SE-iFISH platform is predictive of chemoradiotherapy sensitivity and PFS in colorectal cancer patients.

In conclusion, our study showed that CTCs could be detected in various stages of colorectal cancer using the novel SE-iFISH platform. The sensitivity and specificity were 90.9% and 82.4% using the cutoff value of one cell in 7.5 mL of blood sample. Dynamic monitoring of CTC numbers was able to predict colorectal cancer recurrence.

## MATERIALS AND METHODS

### Patients and sample collection

Forty-four CRC patients and 17 healthy controls were prospectively recruited from the Aviation Hanzhong 3201 Hospital (Shanxi, China). All patients were histologically diagnosed with CRC and underwent radical resection (R0) of primary tumor and thereafter received chemotherapy (see below for details). Patients with preoperative chemoradiotherapy, other concomitant or previous malignancies were excluded. The 17 healthy controls included 11 male and 6 female with age ranging between 18 and 70 years old and were free of colorectal polyps and CRC according to colonoscopy report and with no history of any cancer. This study was approved by ethical committee of the hospital and all participants signed informed consent before their enrolment in the study. All patients underwent surgical resection of CRC lesions followed by chemoradiotherapy (if needed), which began at one month after surgery. All patients were followed up for a median period of 19 months (range, 1 – 43 months). To reveal the clinical significance of CTC, peripheral blood samples were collected from colorectal cancer patients one day before surgery (preoperative), one week after surgery (postoperative one week, P1W), and three months after surgery (postoperative 3 months, P3M).

### Detection of CTCs using SE-iFISH and cellsearch system

Peripheral blood samples (7.5mL) were collected from all participants in an ACD tube (Becton Dickinson, Franklin Lakes, NJ, USA) and subjected to subtraction enrichment of CTCs. The first 1ml blood had been discarded to exclude the merkel cells from the skin. All samples were processed within 48h after collection according to previous studies [23, 29] using the Cytelligen CTC enrichment kit (Cytelligen, San Diego, CA, USA). Briefly, CTCs were enriched from 7.5 mL peripheral blood samples after thoroughly mixed with 3 mL of hCTC separation matrix (Cytelligen) and centrifuged at 450×g for 5 min at the room temperature. Supernatants were then collected and incubated with immunomagnetic particles that were conjugated to a monoclonal antibody anti-leukocyte antigenCD45 at room temperature for 10 min with gentle agitation. The mixture was then subjected to magnetic separation using a magnetic stand (Promega, Madison, WI, USA) to remove leukocytes. The magnetic particle-free solution was spun at 500×g for 2 min at the room temperature and sediment cells were mixed thoroughly with cell fixative from Cytelligen and applied to coated CTC slides for further analysis. The slides were then air-dried at 32°C for 4 h before iFISH. Cells from the above procedures were first immunostained with a monoclonal anti-CD45 antibody conjugated to Alexa Flora 594 (Invitrogen, Carlsbad, CA, USA) and with an anti-PanCK (recognizing CK4, 5, 6, 8, 10, 13, and 18 from Invitrogen) at the room temperature for 2 h, followed by FISH analysis. FISH was performed with Centromere Probe 8(CEP8) spectrum Orange (Vysis, Abbott Laboratories, Abbott Park, IL, USA). Cell nuclei were finally counterstained with DAPI (Invitrogen) and mounted with a mounting medium (Cytelligen). The cells were subsequently subjected to image analysis under a fluorescence microscope (Nikon, Tokyo, Japan). CTCs were defined as CK+/CD45-/DAPI+/CEP8=2, CK+/CD45-/DAPI+/CEP8>2, CK-/CD45-/DAPI+/CEP8>2, whereas CK-/CD45+/DAPI+/CEP8=2 were defined as WBC [23]. Detection of CTCs with the CellSearch system was performed as described previously [16].

### Statistical analysis

All statistical analyses were performed with SPSS 19.0 software (SPSS, Chicago, IL, USA). Association of CTCs positive rates with various parameters was analyzed using Fisher's exact test. Kaplan-Meier survival curves and log-rank test were used to compare progression free survival (PFS). Graphical plots were generated using GraphPad prism version 5 software (GraphPad Software, La Jolla, CA, USA) and OriginPro 8 (OriginLab Corporation, Northampton, MA, USA). A *P* value ≤ 0.05 was considered as statistically significant.

## Abbreviations

EpCAM: epithelial cell adhesion molecule
iFISH: immunostaining fluorescence in situ hybridization
DAPI: 4′,6-diamidino-2-phenylindole
CEP8: Centromere Probe 8
CK: cytokeratin
CRC: Colorectal cancer
CTC: Circulating tumor cells
CEA: carcinoembryonic antigen
CA199: carbohydrate antigen 19-9
EMT: Epithelial-mesenchymal transition.
